# Cranial Computed Tomography Associated with Development of Functional Dependence in a Community-based Elderly Population

**DOI:** 10.2188/jea.12.153

**Published:** 2007-11-30

**Authors:** Eri Tsukishima, Hiroya Saito, Koichi Shido, Gen Kobashi, Gong Ying-Yan, Reiko Kishi, Syuhei Takeuchi, Minehisa Niino, Kiyotaro Kondo, Iwao Sugimura

**Affiliations:** 1 Department of Public Health, Hokkaido University Graduate School of Medicine.; 2 Department of Radiology, Asahikawa Kosei General Hospital.; 3 Department of Social Services, Graduate School of Health Sciences, University of Hokkaido.; 4 Department of Health for Senior Citizens, Hokkaido University Graduate School of Medicine.; 5 Department of Health Examination, Asahikawa Kosei General Hospital.; 6 Faculty of Liberal Arts, University of the Air.; 7 The Director of Asahikawa Kosei General Hospital.; 8 Sapporo City Kiyota Health Center.

**Keywords:** brain atrophy, silent infarct, leukoaraiosis, computed tomography, activities of daily living

## Abstract

Objective: To investigate whether changes at computed tomography (CT) imaging in the ageing brain are associated with future risks for functional dependence. Subjects: 160 residents aged 69 years and older at the cranial CT and were independently living in a rural community in Hokkaido, Japan. Methods: Cranial CT was performed between 1991 and 1993, graded for ventricular enlargement, sulcal enlargement, white matter change, and small infarction. Functional status was reassessed in 1998 in each participant. Multiple logistic regression analysis was performed to estimate the association of CT changes in the ageing brain with development of functional dependence over six years. Results: Functional dependence was found in 19 residents at the second survey. After adjusting for age, sex, medical conditions, and cognitive functioning, small infarction and ventricular enlargement were significantly associated with development of functional dependence (adjusted odds ratio = 9.27 and 4.62). Conclusions: After controlling for age, the age-related changes on cranial CT have significant association on development of functional dependence.

## INTRODUCTION

Brain atrophy, white matter change (leukoaraiosis), and small infarction are frequently detected in healthy old people who have no clinical manifestations, using computed tomography (CT) and magnetic resonance imaging (MRI).

These changes appeared to be associated with cognitive decline^1^^-^^3^^)^ and neurological damage^4^^,^^5^^)^. The psycho-neurological deficit associated with changes in the ageing brain probably cause functional dependence in activities of daily living (ADL), which is one of the primary domains of health-related quality of life^6^^)^.

Several epidemiological studies showed that brain atrophy, white matter change and small infarction were associated with hypertension^7^^-^^9^^)^, cigarette smoking^10^^)^, or coagulation factors^9^^)^.

Our longitudinal study examined healthy old people living in a rural town, Takasu, Japan, to investigate clinical and epidemiological aspects of age-related changes in the brain that appeared on CT. Recently we demonstrated an association between longitudinal blood pressure variability and cerebrovascular changes^11^^)^. In this report, we examined whether the participants had developed functional dependence over six years, and investigated the association of changes in the ageing brain on subsequent development of functional dependence.

## SUBJECTS AND METHODS

### Study Participants

The potential participants for this study were all elderly people in Takasu, which was an agricultural town in a northern part of Hokkaido, Japan, with around 8,000 residents. Cranial CT scanning was planned for the entire elderly population aged 69 years and older, and performed between December 1991 and February 1993 for 193 persons, among a total of 331 persons. Participants for the current study included the 160 residents, 84 men and 76 women, aged 73.8± 3.4 years (mean±standard deviation (SD)), who (a) were independently living at their home at the time of CT scanning, requiring no assistance for getting outside and their ADL; (b) had no histories of stroke; (c) had no definite abnormalities on the cranial CT scanning, such as intracranial tumor, extradural hydroma, or large infarction (> 30mm).

### Cranial CT and Baseline Data

**Figure 1.**
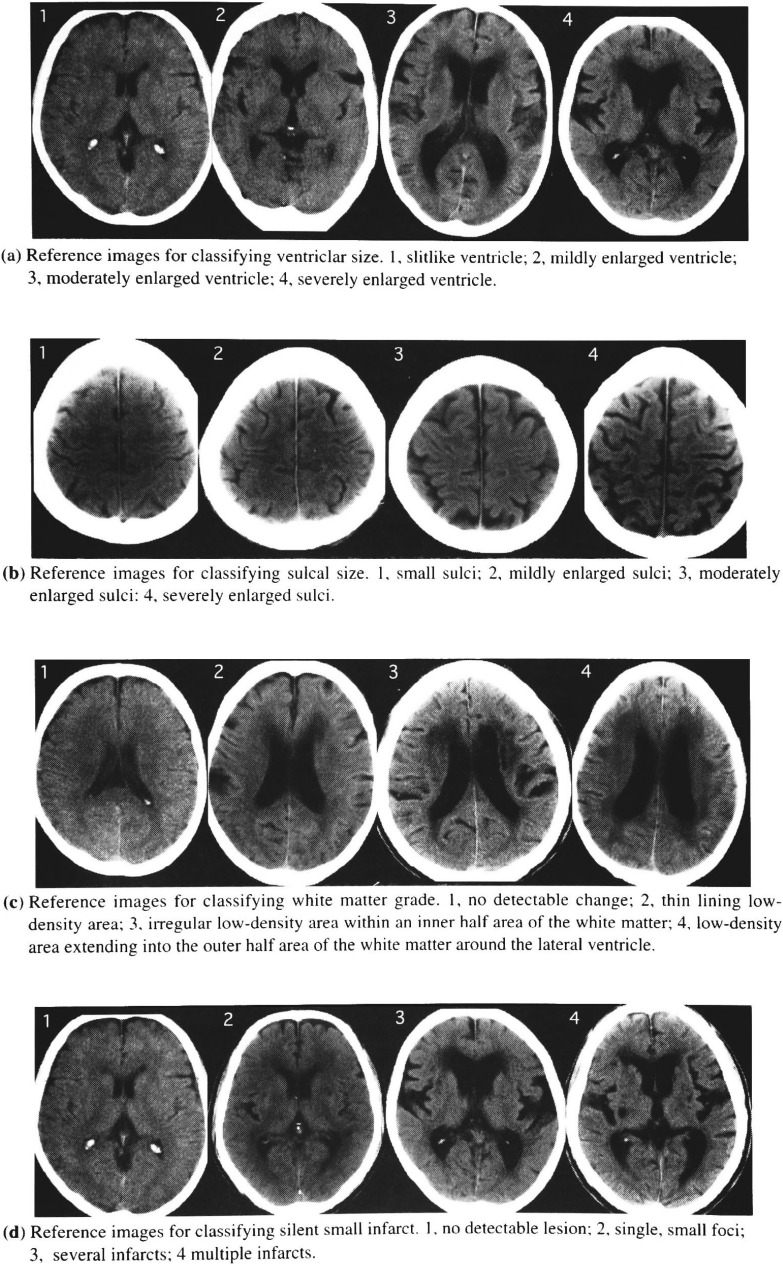
Predefined visual standards for classifying changes in the ageing brain detected on CT.

Axial CT scans were obtained with 10-mm-thick sections. Two experienced radiologists, who were blinded to the clinical information, graded all the CT scans. Global brain atrophy, as reflected by ventricular and sulcal size, white matter change, and small infarction, were graded according to predefined visual standards (Figure 1). Ventricular enlargement and sulcal enlargement were assessed by grading the size of lateral ventricle and sulcal size. White matter change was assessed by grading the extent of low-density area in the periventricular white matter. Small infarction was assessed by the presence of small round low-density foci less than 30 mm in diameter. The reference standards for classifying age-related changes are ranged from no or minimal change (grade 1) to severe change (grade 4). The intrarater reliabilities of the classifications were within acceptable range (Kappa = 0.85 to 0.96).

On the same day of CT scanning, baseline characteristics were obtained for all participants. Demographic characteristics, lifestyles, self-reported global daily function, and previous diagnosis were obtained via interview by trained public health nurses. Global daily function was rated into 5 categories by the national standard for classifying tendency to bed-ridden state: grade 0 was defined as those completely independent both in getting outside from home and in ADL; grade J was as those who could get outside without assistance while they had some difficulty; grade A was defined as those who were independent in basic ADL (eating, dressing, and using the toilet), but unable to get outside without assistance; grade B was defined as those who needed some assistance in their basic ADL; and grade C was defined as so-called ‘bed-bound’, those who needed assistance in all of the basic ADL, and who were unable to leave their bed without assistance. Thus the participants who reported their global daily function as grade 0 and J were included in the following analysis. All participants were also interviewed whether they were previously diagnosed as; heart disease, diabetes mellitus, hypertension, respiratory disease and arthritis. Physical and neurological examinations performed on the same day included blood-pressure measurement and The Kana Pick-out Test. The Kana Pick-out Test, as a measurement of prefrontal lobe functioning, was developed to assess early cognitive impairment in the healthy elderly persons in Japan^12^^)^.

### Outcome Measurement

In July 1998, the second survey examined functional status of all the participants, who were rated as independent in their ADL at the time of the cranial CT. The primary outcome was development of functional dependence, defined as admission to a nursing facility before July 1998, or having a new disability in getting outside or in ADL at the second survey.

Admission to nursing facilities such as nursing home and hospital for long-term care, for residents who had difficulty in daily life, was ascertained from the residential health records of the town. Functional status of other responders was examined by self-reported questionnaire. Global daily function was rated by the national standard for classifying tendency to bed-ridden state as at the time of CT scanning. The participants who reported their global daily function as grade A, B, and C at the second survey were rated as those with development of functional dependence. When available, proxy responses were used for participants who were unable to complete this assessment.

### Statistical Analysis

Each grade for the changes in the brain was dichotomized to avoid cells with small numbers. For example, ventricular enlargement graded 3 and 4 was compared with graded 1 or 2. Associations between CT findings and baseline characteristics were analyzed cross-sectionally by chi-square tests and Student’s t-tests. Differences between residents with and without the development of functional dependence were analyzed by Student’s t-test for continuous variables and chi-square test for categorical variables.

Multiple logistic regression analysis was used to estimate association of changes in the ageing brain with development of functional dependence. In the final model, changes in the ageing brain were compulsorily entered, and potential confounding factors, such as age, sex, and medical conditions were considered and selected into the model by stepwise analysis. We used the computer software package Statistical Analysis System (SAS) ver. 6.

## RESULTS

Among 160 healthy old residents, 17 residents had ventricular enlargement (grade 3, 4), 50 had sulcal enlargement (grade 3, 4), 28 had white matter change (grade 2, 3, 4), and 22 had small infarction (grade 2, 3, 4). These changes were associated with older age, higher blood pressure, and history of hypertension. Those who had small infarction had lower score for The Kana Pick-out Test (Table 1).

**Table 1.** Cross-sectional association of variables at the first survey with cranial CT findings in a healthy elderly population.

grades	Ventricular size		Sulcal size		White matter change		Small infarction	
			
	1,2	3, 4	1,2	3, 4	1	2, 3, 4	1,2	2, 3, 4

Number of subjects	n=143	n=17	n=110	n=50	n=132	n=28	n=138	n=22
Age at CT (year, mean±SD)	73.6±3.4	75.4±2.6 ^a^	73.1±3.1	75.4±3.4	73.7±3.5	74.3±2.9	73.5±3.3	75.3±3.2 ^a^
Male sex (%)	74 (51.8)	10 (58.8)	55 (50.0)	29 (58.0)	68 (51.5)	16 (57.1)	72 (52.2)	12 (54.6)

Systolic blood pressure (mmHg, mean±SD)	145.2±20.6	158.2±25.1 ^a^	146.9±22.3	146.0±19.7	144.8±19.9	154.8±26.7	145.6±19.9	152.6±29.2
Diastolic blood pressure (mmHg, mean±SD)	79.9±13.5	87.6±13.0 ^a^	80.5±14.1	81.3±12.6	80.0±13.7	84.1±13.0	79.7±13.4	87.2±13.5 ^a^
Kana Pick-out Test (mean±SD)	15.8±10.4	12.9±8.5	15.5±9.1	15.4±12.5	15.8±10.7	13.8±7.9	16.3±10.3	10.0±8.4 ^a^

Previously diagnosed as;								
Hypertension (%)	56 (39.4)	8 (47.1)	42 (38.5)	22 (44.0)	47 (35.9)	17 (60.7) ^b^	50 (36.5)	14 (63.6) ^b^
Heart diseases (%)	34 (23.9)	7 (41.2)	24 (21.8)	17 (34.0)	31 (23.7)	10 (35.7)	31 (22.5)	10 (47.6) ^b^
Diabetes (%)	17 (12.0)	4 (23.5)	13 (11.9)	8 (16.0)	18 (13.7)	3 (10.7)	17 (12.4)	4 (18.2)
Respiratory diseases (%)	10 (7.1)	2 (11.8)	10 (9.2)	2 (4.0)	10 (7.6)	2 (7.4)	12 (8.7)	0 (0.0)
Arthritis (%)	64 (45.1)	9 (52.9)	53 (48.2)	20 (40.0)	62 (47.0)	11 (40.7)	60 (43.5)	13 (61.9)

Cranial CT findings								
Ventricular enlargement (%)	-	-	8 (7.3)	9 (18.0) ^b^	10 (7.6)	7 (25.0) ^c^	12 (8.7)	5 (22.7) ^b^
Sulcal enlargement (%)	41 (28.7)	9 (52.9) ^b^	-	-	40 (30.3)	10 (35.7)	41 (29.7)	9 (40.9)
White matter change (%)	21 (14.7)	7 (41.2) ^c^	18 (16.4)	10 (20.0)	-	-	16 (11.6)	12 (54.6) ^c^
Small infarction (%)	17 (11.9)	5 (29.4) ^b^	13 (11.8)	9 (18.0)	10 (7.6)	12 (42.8) ^c^	-	-

Data are mean±SD or frequency (percent). ^a^ p<.05 using Student’s t test; ^b^ p<.05; ^c^ p<.01 using chi-square test.

The second survey in 1998 showed that among 160 residents, 13 (8.0%) died without admissions to nursing facilities before death and 16 (10.0%) were missing because of refusal, removal, and inappropriate assessment of function for an acute phase of their disease, leaving 131 participants in the following analysis. Persons who had died had higher age than those in the analysis, and severe ventricular enlargement, sulcal enlargement, and small infarction on cranial CT. Persons who had no outcome data had lower frequency in heart disease than in the analysis, and there were no significant differences in cranial CT findings between those without outcome data and those in the analysis.

Assessment of functional status showed that 112 (69.1%) residents were independent at home, 19 (11.7%) had developed functional dependence over six years. In the 19 residents, four had been admitted in nursing facilities prior to the survey, and 15 lived at home but required assistance for daily life.

Baseline characteristics and cranial CT were compared between residents with and without development of functional dependence over six years (Table 2). In subjects who developed functional dependence, baseline prevalence of ventricular enlargement, sulcal enlargement, and small infarction were significantly higher than in subjects without dependence. Of the nineteen subjects who developed functional dependence in six years, eight (41.2 %) had small infarction on their cranial CT, otherwise, only 9.0 % of subjects who did not develop functional dependence later had small infarction in their cranial CT. As for white matter change, residents with detectable change seemed to have a higher risk of functional dependence, however, this tendency was not significant. Mean age was significantly higher in residents with functional dependence than without (p<0.0002). Characteristics of lifestyles and medical conditions were not significantly different between residents with and without functional dependence.

**Table 2.** Comparison of cranial CT findings and baseline characteristics between the elderly residents with and without development of functional dependence over six years.

	Functionally independent	Functionally dependent	p-value
	
	(n=112)	(n=19)	
Cranial CT findings; grades			
Ventricular enlargement; 3, 4	8 ( 7.2)	5 (26.3)	0.01
Sulcal enlargement; 3,4	30 (26.8)	10 (52.6)	0.02
White matter change; 2, 3, 4	19 (17.2)	4 (21.0)	0.67
Small infarction; 2, 3, 4	10 ( 9.0)	8 (41.2)	0.001

Demographic character			
Age at the second survey, year	79.4 ± 3.3	82.5 ± 3.3	0.0002
Age at CT, year	73.3 ± 3.2	76.3 ± 3.3	0.0002
Male sex	56 (50.0)	12 (63.2)	0.329

Medical history at CT			
Systolic blood pressure, mmHg	146.2 ± 20.8	149.5 ± 20.6	0.52
Diastolic blood pressure, mmHg	81.1 ± 14.1	82.8 ± 14.1	0.62
Hypertension	42 (37.8)	8 (42.1)	0.8
Heart diseases	28 (25.2)	8 (42.1)	0.17
Diabetes	15 (13.4)	4 (21.0)	0.48
Respiratory diseases	9 ( 8.2)	2 (10.5)	0.67
Arthritis	56 (50.0)	8 (42.1)	0.62
Kana Pick-out Test	16.2 ± 10.4	12.9 ± 9.5	0.19

Data are number (%) or mean ± SD.

Differences were analyzed by chi-square test and Student’s t-test.

Logistic regression analysis showed the association between changes in the ageing brain and development of functional dependence (Table 3). In univariate analysis, ventricular enlargement, sulcal enlargement, and small infarction had significant association with functional dependence (crude odds ratio (OR) = 4.64, 3.04, and 7.42; for ventricular enlargement, sulcal enlargement, and small infarction, respectively). White matter change did not have significant association with development of functional dependence.

**Table 3.** Odds ratios for development of functional dependence over six years associated with changes in the ageing brain : logistic regression analysis

	Univariate model	Age-adjusted model	Multivariate model ^a^
Ventricular enlargement	4.64	3.69	5.26
	(1.33, 16.19)	(0.85, 16.08)	(1.03, 29.02)

Sulcal enlargement	3.04	1.66	2.28
	(1.13, 8.20)	(0.51, 5.39)	(0.64, 8.48)

White matter change	1.31	0.46	0.28
	(0.39, 4.37)	(0.08, 1.97)	(0.04, 1.42)

Small infarction	7.42	7.73	24.00
	(2.42, 22.70)	(2.09, 31.46)	(4.76, 157.73)

Data are odds ratios (95% confidence interval).

^a^ Significant variables (p<0.20) among age, sex, blood pressure, Kana Pick-out test and previous diagnosis were entered.

Age at the second survey, diastolic blood pressure, respiratory diseases, arthritis were stayed in the final model by stepwise selection (p<0.20).

A step-wise analysis was used to select variables, such that, age at the second survey, diastolic blood pressure, respiratory diseases, and arthritis remained in the final multivariate model (p<0.20), whereas the Kana Pick-out Test did not remain significant. The final model showed small infarction had the highest odds ratio (adjusted OR, 95% confidence interval (CI); 24.00, 4.76-157.73), and ventricular enlargement also had significantly high odds ratio (adjusted OR, 95% CI; 5.26, 1.03-29.02). Adjusted OR of sulcal enlargement deteriorated to 2.28 (95% CI, 0.64-8.48) in the final model.

## DISCUSSION

The present study examined a community-living elderly population for development of functional dependence over six years, and showed that small infarction and ventricular enlargement on cranial CT scanning were significantly associated with functional dependence, after adjusting for age, sex, and medical conditions. Multivariate analysis showed subjects with small infarction had 24.0 times as high risk for functional dependence as those without, and subjects with ventricular enlargement had 5.26 times high. These radiographical changes seemed to express earlier stage of functional dependence in the old people.

Several cross-sectional studies showed that age-related changes were associated with cognitive decline and neurological deficit. Small infarction was associated with several disabilities in neuropsychological tests^3^^,^^5^^)^, depressive mood^13^^)^, and future risks for clinical stroke^14^^)^. Ventricular enlargement was associated with poorer performances in cognitive function tests^1^^)^. A longitudinal study using volumetric analysis of the brain showed that volume loss of the hippocampus and temporal lobe in healthy elderly persons predicted development of dementia^15^^)^. In our study, cranial CT findings were significantly associated with development of functional dependence, even when the Kana Pick-out Test was taken into account, which was one of the cognitive measurements. The Kana Pick-out Test was suggested to be sensitive for assessment of some aspects of cognitive functioning, since the score had statistically significant associations with cranial CT, though Hasegawa’s Dementia Scale Revised (HDS-R) did not, in 58 of our study participants (data not shown). Our findings are consistent with the interpretation that cognitive decline and neurological dysfunction can affect ADL, and changes in the ageing brain consequently have an association with future development of functional dependence in the older people.

In contrast to ventricular enlargement and small infarction, the present study showed that the effects of white matter change and superficial brain atrophy, as reflected by sulcal enlargement, did not remain significant after controlling for age in the multivariate analysis. Moreover, cognitive function score in the follow-up assessment was lower in residents with these changes, but the difference was not significant (data not shown). Several cross-sectional studies showed that white matter hyperintensities on MRI were associated with cognitive decline^1^^)^, and neurological dysfunction^4^^)^. Our longitudinal study, however, differed for using computed tomography, which might be less sensitive for defining white matter change than MRI, and thus the disparity in the results, although the reliabilities of the four-point scale were of acceptable range (Kappa = 0.85 to 0.96).

Multiple factors are related to the changes in the ageing brain, such that findings on neuro-imaging technique have high variance in old people. For example, average brain volume in old people were lower than in young people in healthy volunteers as expected^16^^)^, however, in the same population, variance of brain volume in old people was evidently higher than in young people. In addition, hypertensive subjects had more atrophied brain than normotensives in the same age groups^7^^)^. White matter change and small infarction were associated with hypertension^8^^,^^9^^)^, cigarette smoking^10^^)^, or coagulation factors^9^^)^. These findings suggest that ageing itself and other various factors have combined effects on the changes in the ageing brain. More efforts to prevent these changes need to be performed to improve quality of life in the old people.

Functional status is one of the most important domains of quality of life in old people^17^^)^. Functional decline was found to be associated with age, smoking status, marital status^18^^)^, chronic diseases^19^^)^, cognitive problems^20^^)^, and self-rated health^21^^)^. Similarly in our study population, functional dependence was caused by multiple factors; however, the proportion of subjects with small infarction was 41.2 % of the subjects who developed functional dependence. Moreover, more than 90 % of the subjects who did not develop dependence did not have small infarction at the baseline CT. These values support previous reports, which showed cerebrovascular diseases, and cognitive decline had important effects on disability when subjects had comorbidity as well^19^^)^. In addition, baseline vulnerability also increased effects combined with acute hospital event on the development of functional dependence^22^^)^.

One major limitation of this study is that functional dependence was assessed only once such that we could not analyze for time effects. Misclassification associated with the outcome assessment may have occurred because it is impossible to differentiate between persons who had been consistently independent and those who had just recovered before the assessment. Previous reports showed that some elderly people had recovered from their dependence during two or three years^18^^,^^20^^)^. Several assessments in future analyses should, therefore, be considered to clarify how neuro-anatomical changes in old people influenced their daily function.

In conclusion, the present longitudinal study showed that small infarction and ventricular enlargement have significant association with the development of functional dependence. Old persons who have these radiographical changes should be considered to be in their earlier stages of functional dependence, and focused to improve quality of life in their later life. And more efforts to prevent age-related brain changes, such as controlling hypertension and cigarette cessation, are certainly needed.
